# Tail suspension is useful as a sarcopenia model in rats

**DOI:** 10.1186/s42826-020-00083-9

**Published:** 2021-01-13

**Authors:** Akira Nemoto, Toru Goyagi

**Affiliations:** Department of Anesthesia and Intensive Care Medicine, Akita University Graduate School of Medicine, 1-1-1, Hondo, Akita, 010-0843 Japan

**Keywords:** Sarcopenia, Muscle atrophy, Rat, Hind limb unloading, Tail-suspension, Skeletal muscle mass

## Abstract

**Background:**

Sarcopenia promotes skeletal muscle atrophy and exhibits a high mortality rate. Its elucidation is of the highest clinical importance, but an animal experimental model remains controversial. In this study, we investigated a simple method for studying sarcopenia in rats.

**Results:**

Muscle atrophy was investigated in 24-week-old, male, tail-suspended (TS), Sprague Dawley and spontaneously hypertensive rats (SHR). Age-matched SD rats were used as a control group. The skeletal muscle mass weight, muscle contraction, whole body tension (WBT), cross-sectional area (CSA), and Muscle RING finger-1 (MuRF-1) were assessed. Enzyme-linked immunosorbent assay was used to evaluate the MuRF-1 levels. Two muscles, the extensor digitorum longus and soleus muscles, were selected for representing fast and slow muscles, respectively. All data, except CSA, were analyzed by a one-way analysis of variance, whereas CSA was analyzed using the Kruskal-Wallis test.

Muscle mass weight, muscle contraction, WBT, and CSA were significantly lower in the SHR (*n* = 7) and TS (*n* = 7) groups than in the control group, whereas MuRF-1 expression was dominant.

**Conclusions:**

TS and SHR presented sarcopenic phenotypes in terms of muscle mass, muscle contraction and CSA. TS is a useful technique for providing muscle mass atrophy and weakness in an experimental model of sarcopenia in rats.

## Background

Sarcopenia, a major geriatric disorder that presents with symptoms, such as loss of muscle mass and strength [1], and involves a histological reduction in muscle fiber size and number. There are two types of sarcopenia: primary sarcopenia that is solely due to unexplained aging, and secondary sarcopenia that is caused by one or more factors associated with chronic disease, nutrition, and physical activity. Since its prevalence increases with age, sarcopenia affects 26.8% of men and 22.6% of women over 64 years of age [2]. Sarcopenia involves muscle atrophy and carries a high mortality rate [3, 4]. Therefore, understanding its clinical development to be able to increase patient’s quality of life is of the highest importance.

In humans, a recent statement defined sarcopenia as having four aspects: walking speed of < 1 m/sec, walking distance of < 400 m during a six-minute walk test, a lean appendicular mass, corrected for height squared of 2 standard deviations or more below the mean of healthy persons between 20 and 30 years of age of the same ethnic group [5]. Some studies reported on an experimental sarcopenia model, but there is a lack of consensus regarding using a rodent model. Aged animals, accelerated senescence, unloading and immobilization have been recognized as sarcopenia models [1]. Muscle atrophy due to disuse has been proposed to develop into sarcopenia [6]. However, an effective therapy for sarcopenia has not yet been identified. Creating a simple animal experimental model of sarcopenia, other than aging, is essential for studying the prevention of diseases caused by sarcopenia. Since hypoactivity and metabolic syndrome are highly associated with sarcopenia [7, 8], this study aimed to examine a suitable experimental model in rats based on the association of hypoactivity and metabolism with sarcopenia. Metabolic syndrome can lead to type 2 diabetes mellitus (DM), which induces insulin resistance, hypertension, and other conditions. In addition, DM and insulin resistance cause low skeletal muscle mass and muscle weakness [9, 10]. Spontaneously hypertensive rats (SHR) are widely used in hypertension and insulin-resistance models [11]. For those reasons, we selected SHR for our metabolic model. In addition, rats with tail suspension (TS) were used to represent a low activity model, which involves decreased hind limb function by tail suspension. Low activity models involve candidates being maintained in limited activity, such as unloading (removal of weight) and immobilization (to reduce muscle mass). Previous unloading models attenuated the hind limb contraction using various techniques, such as using a body harness, surgical pin or TS [12]. These hind limb unloading models are widely used to understand osteopenia and muscle atrophy. TS, in particular, is a non-invasive and simple method. Therefore, in this experiment, we compared TS and SHR with control rats, to determine whether they exhibited skeletal muscle loss.

## Results

### Extensor digitorum longus and soleus muscle weights

The mean extensor digitorum longus (EDL) muscle weight was lower in the TS and SHR groups than in the control group (Table 1). Similarly, the weights of the soleus (SOL) muscle were lower in the TS and SHR groups than in the control group (Table 1). However, there is no significant difference in muscle weight, adjusted for each body weight (Table 1).

**Table 1:** Skeletal muscle mass weight and muscle to body weight

	control	TS	SHR
body weight (g)	658 ± 30	575 ± 22 *	431 ± 10 *
EDL muscle (g)	0.33 ± 0.01	0.28 ± 0.01 *	0.19 ± 0.01 *
EDL/BW (g/kg)	0.51 ± 0.03	0.49 ± 0.03	0.44 ± 0.02
SOL muscle (g)	0.98 ± 0.11	0.72 ± 0.03 *	0.61 ± 0.06 *
SOL/BW (g/kg)	1.53 ± 0.19	1.28 ± 0.08	1.41 ± 0.15

*EDL muscle* Extensor digitorum longus muscle, *SOL muscle* Soleus muscle; *SHR* Spontaneously hypertensive rat, *TS* Tail suspension, *BW* Body weight. *P < 0.05 vs. control group, values = mean ± SEM (*n* = 7 per group)

### Muscle contraction

**Fig. 1:**
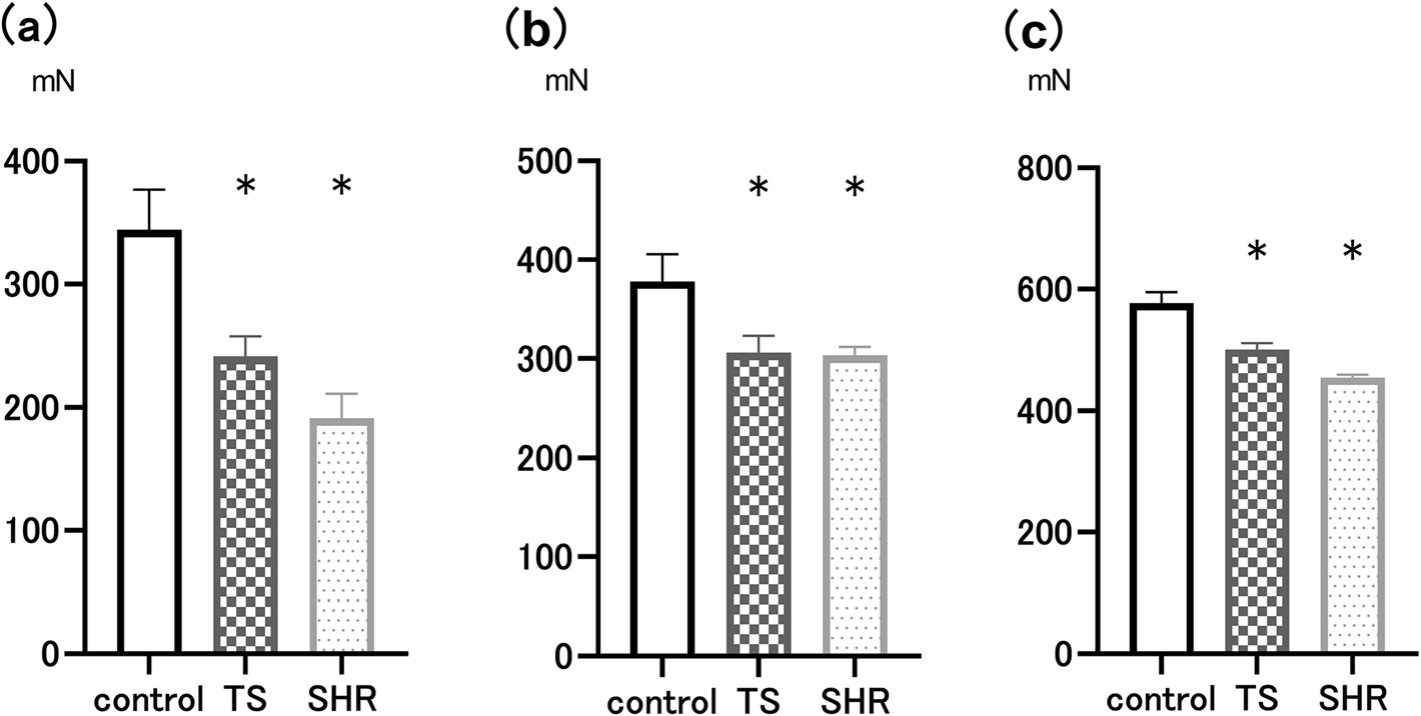
Skeletal muscle contraction. **a** Contraction of the extensor digitorum longus (EDL) muscle. **b** Contraction of the soleus (SOL) muscle. **c** Whole body tension (WBT). The twitch contraction force of EDL muscle in the TS and SHR groups were weaker than that of the control group. Similarly, those of the SOL muscle in the TS and SHR groups indicated significant reduction compared to the control group. The WBT10 in the TS and SHR groups significantly reduced compared with the control group. The data are represented as mean ± SEM (*n* = 7 per group). SHR, spontaneously hypertensive rat; TS, tail suspension. *P < 0.05 vs control group

The twitch force of the EDL muscle caused by electric stimulation differed significantly between the control and the two test groups. The contraction force of EDL muscle in the TS and SHR groups decreased by 30% (*p* = 0.01) and 45% (*p* < 0.01), respectively, compared with the control group (Fig. 1a). Regarding SOL muscle, the contraction force in the TS and SHR groups significantly decreased by 19% (*p* = 0.03) and 20% (*p* = 0.03) compared to the control group, respectively (Fig. 1b), whereas there is no difference in contraction between the TS and SHR groups. These results indicate that the TS group exhibited a reduced contractile force and SHR rats exhibited lower-limb muscle weakness.

### Whole body tension

The result of the whole body tension (WBT) measurement is indicated in Fig. 1c. The WBT10 in TS and SHR was significantly lower than that in the control group, which represents the average of the top 10 pulling forces. However, there was no significant difference when adjusted for each body weight.

### Cross-sectional area

**Fig. 2:**
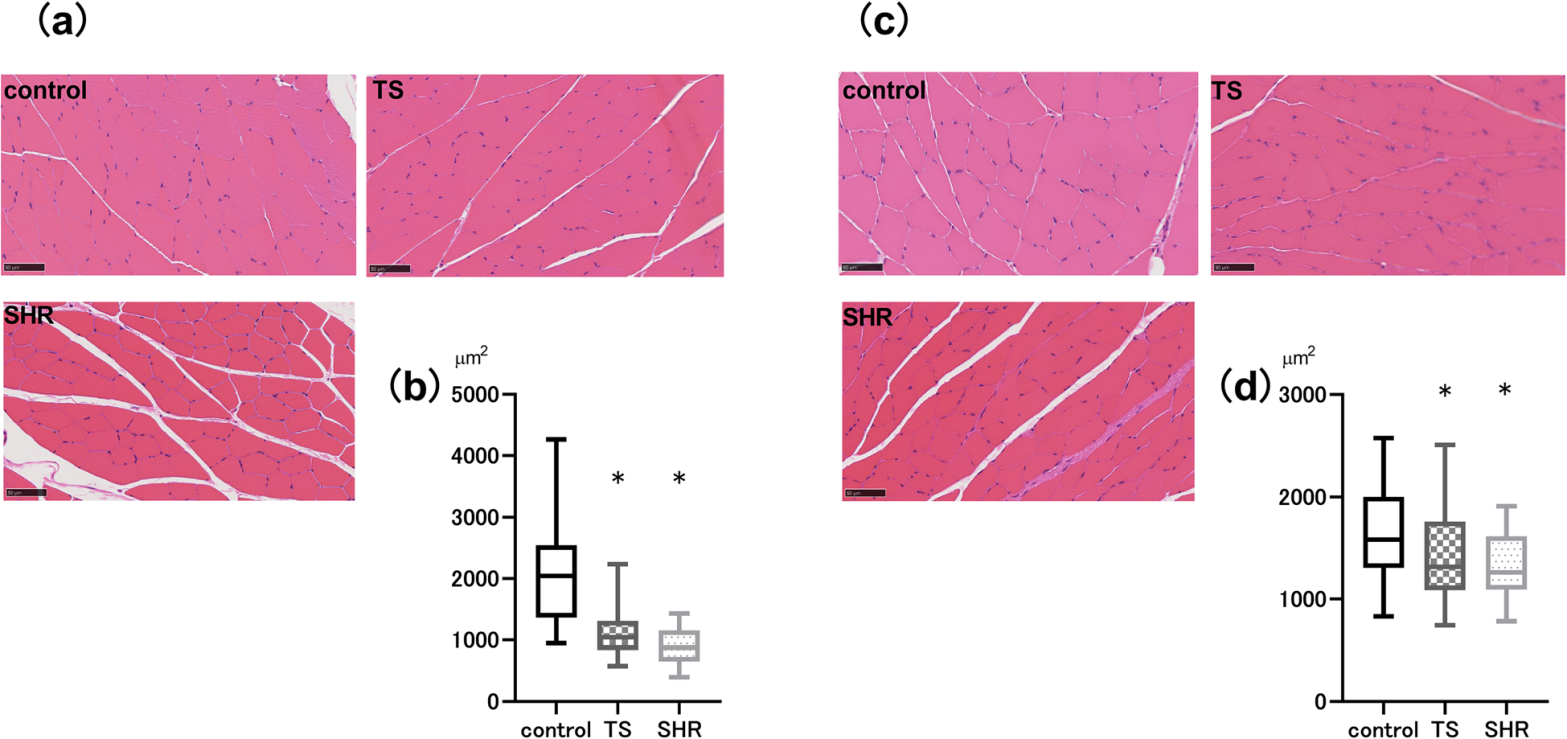
The muscle fiber size; cross sectional area (CSA). **a** Representative histological digital images stained by hematoxylin and eosin (HE) of extensor digitorum longus (EDL) muscle, **b** Comparison of EDL muscle fiber size, **c** Representative histological digital images stained by HE of soleus (SOL) muscle, **d** Comparison of EDL muscle fiber size. The reference bar in histological images is equal to 50 μm. The CSA of EDL and SOL muscles in both TS and SHR groups were significantly smaller than that in the control group. Box-and-whisker-plots show 95% confidence interval, 25th percentiles and 75th percentiles, and medians (*n* = 7 per group). TS, tail suspension, SHR, spontaneously hypertensive rat, *P < 0.05 vs control group

Fig. 2a and c show representative muscle fibers from the TS, SHR and control groups. The cross-sectional area (CSA) of the EDL and SOL muscles were significantly decreased in the TS and SHR group versus the control group (Fig. 2b, d). The median CSA of the EDL muscle in the TS and SHR groups were significantly smaller than that of the control group (Fig. 2b). Similarly, the median CSA of the SOL muscle was smaller in the TS and SHR groups than in the control group (Fig. 2d).

### Muscle RING finger-1

**Fig. 3:**
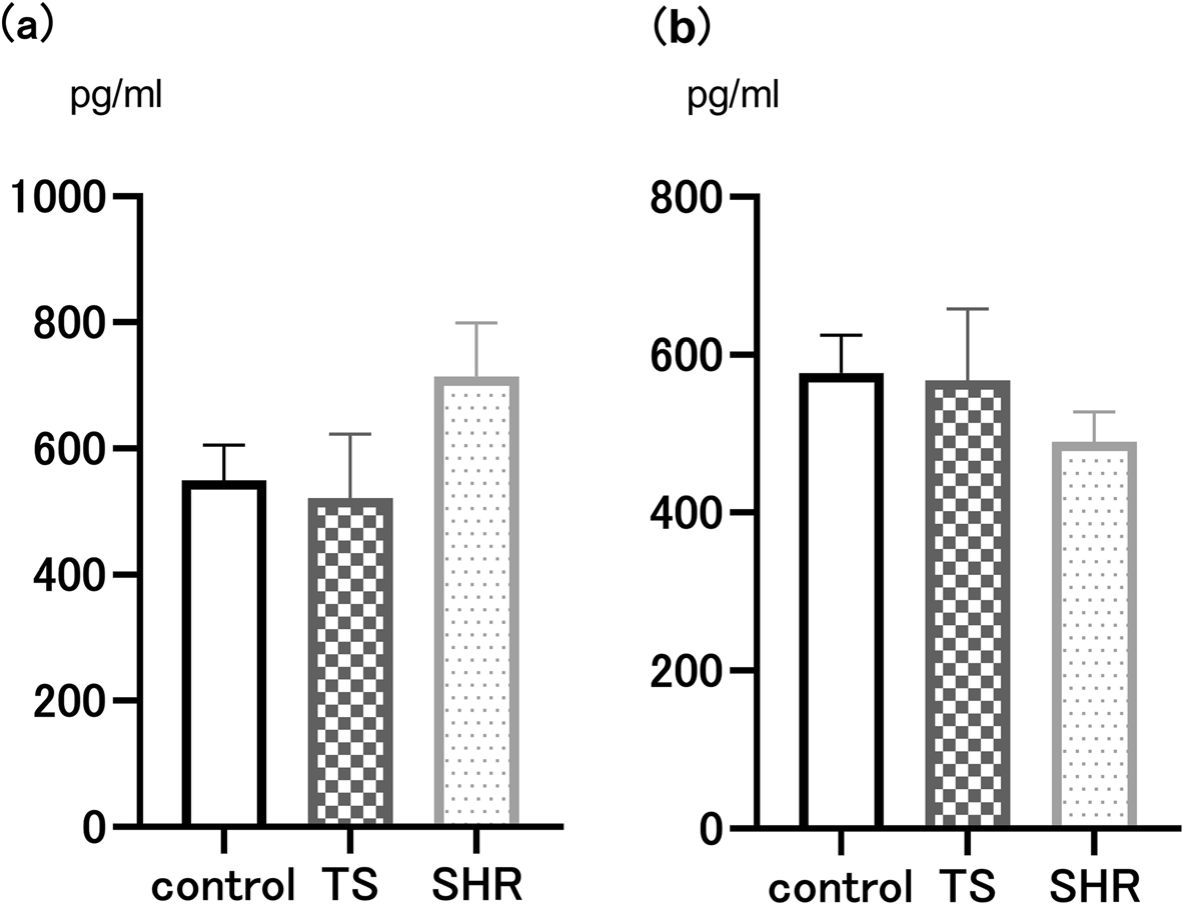
Muscle RING finger-1 (MuRF-1). **a** Concentration of MuRF-1 in extensor digitorum longus (EDL) muscle, **b** Concentration of MuRF-1 in soleus (SOL) muscle. Quantitative analysis was conducted by enzyme-linked immunosorbent assay. In both EDL and SOL muscles, there were no significant differences among three groups. The data are represented as means ± SEM (*n* = 5 per group). TS, tail suspension, SHR, spontaneously hypertensive rat, *P < 0.05 vs control group

Muscle RING finger-1 (MuRF-1) levels in the EDL and SOL muscles did not significantly increase in the TS or SHR groups (*n* = 5) compared to the control group (Fig. 3a, b).

## Discussion

We found that TS and SHR exhibited induced muscle mass atrophy compared to the control rats, whereas MuRF-1 expression did not differ among the groups.

Although similar weight losses of the muscles, EDL and SOL muscles, were seen in the TS and SHR groups, the CSA reduction was greater in the EDL muscle than in the SOL muscle. Sarcopenia is a major geriatric disorder that involves loss of muscle mass and strength [1]. Age induces the loss of type 1 and 2 muscle fibers, but reduces CSA with a dominant effect on type 2 fibers [13]. Therefore, TS and SHR presented phenotypes closer to sarcopenia in terms of muscle mass weight and CSA.

TS is also a simple method used in gravitational unloading models for experimental spaceflights, but its suspension periods remain controversial. The TS method in this study was shorter and easier than those identified in other studies, which are typically as long as 16 days or even 9 weeks [14, 15]. TS leads to hind limb unloading, a method similar to a low skeletal muscle model [16]. It was revealed that four consecutive weeks of hind limb unloading reduced contractions and muscle mass in both fast and slow muscle fibers [17]. TS indicated the same reduction despite a 50% shorter period of hind limb unloading. A previous study has shown that the skeletal muscle atrophy starts during short-term unloading (at 2–4 days) [18] and that the SOL muscle atrophied to nearly half its original size in about 2 weeks [19]. Although a recent study indicated that 7 days of hind limb suspension was enough to reduce the SOL muscle weight in Wistar rats, the contraction and weight of EDL muscle were not measured [20]. The TS fixation method was simple because it involved the suspension of the tail only, whereas previous studies immobilized hind limbs by applying a cast [21]. The findings of this study differ from those of previous similar studies in that there was a reduction in the contractile force and the WBT, and muscle atrophy after 2 weeks of TS. The findings support the need for an additional study to investigate a one-week model of TS as being sufficient for the experimental sarcopenia model.

High calorie food intake accelerates the development of sarcopenia [22, 23]. It also induces metabolic syndrome, DM, insulin resistance, and hypertension. One study showed that about six to seven months old SHR had decreased muscle (as measured in a cross-sectional area per unit weight), weakness and decreased fast muscle components compared to wild-type Wistar Kyoto rats, but with no difference in muscle mass [24]. Adipose tissue is correlated with muscle atrophy [25]. The SHR, in our study, also indicated skeletal muscle atrophy of the EDL and SOL muscles. Thus, the SHR is likely to become a standard as a sarcopenia model.

Ubiquitin-proteasome system plays an important role in intracellular protein degradation [26, 27], accounting for about 80% of the degradation. MuRF-1 and muscle atrophy F-box (MAFbx), muscle-specific E3 ubiquitin ligases, act as markers for muscle-specific ubiquitin-proteasome system. MuRF-1 and MAFbx are regulated by transcription factors, such as forkhead box O (FOXO). Phosphorylated FOXO attenuates the expression of MuRF-1, whereas FOXO promotes it. However, in unloaded rodents, the protein synthesis of MuRF-1 increased in an early stage of suspension [28]. Therefore, no significant increase in MuRF-1 was found in the TS and SHR in this study. Further research is needed to clarify this observation in the TS model.

There are some limitations to this study. First, we examined only twitch contraction without tetanic force. However, in age-related skeletal muscle atrophy, which is represented by sarcopenia, type 2 muscle fibers decline significantly more than type 1 muscle fibers [13]. The electrical stimulation conditions in this experiment preferentially influenced type 2 muscle fibers. This stimulation could underestimate the actual SOL muscle contraction, which mostly consists of type 1 fibers. Although the stimulation provides the principal information, further studies are needed to reveal the effect on type 1 muscle fiber contractions. A limitation of the histological analysis was the use of only one staining method, hematoxylin and eosin (H & E); another staining, such as NADH tetrazolium reductase staining could independently assess type 1 and 2 fibers. Second, the rats used were younger than those in other comparative studies. Aged-rats may be desirable for use in a sarcopenia model because the condition is promoted by aging; however, older rats must be kept for a long time, at a higher cost. This model does not always exhibit an ideal phenotype; therefore, it is somewhat difficult to use as a natural model. Third, significant contraction of EDL muscle was observed compared to that of SOL muscle. This difference could be due to the type of electrical stimulation and whether the muscle fibers are anti-gravity muscles or not. Further experiments are required to confirm if this difference is statistically significant.

Several recent studies have examined sarcopenic models in rodents; however, it is necessary to develop simple models to further understand sarcopenia. Here, we have presented a more simplistic model than in previous studies, using simpler candidates. The SHR are specialized and more expensive compared to normal rats. However, this study indicates that TS can induce sarcopenia in only 2 weeks, even in normal rats.

## Conclusions

TS can induce muscle atrophy and weakness to provide a sarcopenia model in rats.

## Methods

### Animals and experiment

This study was approved by the Institution Animal Care and Use Committee of the Akita University, Akita, Japan (a-1-3012).

**Fig. 4:**
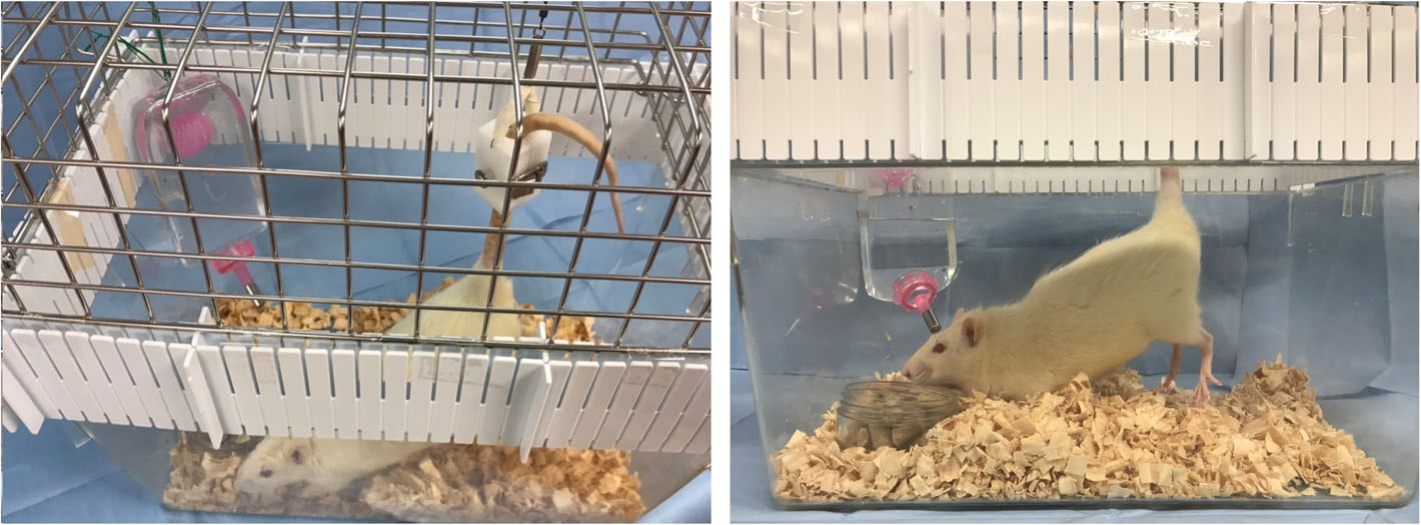
Tail suspension in this study. Pictures of tail suspension. Rats in the tail suspension group were suspended by their tail using a clip for rats. They were allowed to freely access food without touching the ground

In this experiment, 24-week-old male Sprague Dawley (SD) rats (CLEA Japan, Tokyo, Japan) and SHR (SLC Inc., Shizuoka, Japan) were selected. All rats were housed, singularly in cages with controlled temperature (23 ± 3 °C) and humidity (55 ± 15%) on a 12-h dark-light cycle (dark phase from 19:00 to 7:00). All experiments were conducted during the day. We divided the rats into three groups: control, TS, and SHR (*n* = 7 each). SD rats were divided into control and TS groups. The TS group was suspended by the tail using a clip (Yamashita Giken Inc., Tokushima, Japan) to prevent them from touching their hind limbs on the ground, for two consecutive weeks (from 22 until 24 weeks of age) with free access to food and water (Fig. 4). Tape was adhered to the rat’s tail and cushioning added before the suspension line and the clip was fitted. The thickness of the cushioning was adjusted to prevent tail ischemia. Rats who were about to climb their own tails were excluded from the analysis, since that behavior could cause suspension failure. The control and SHR groups were housed in a normal environment.

### Skeletal muscle contractile function

We assessed the contractile force of the fast and slow muscles using sciatic nerve electrical stimulation. The EDL muscle and SOL muscle were selected as examples of fast and slow muscles, respectively. A fast muscle is mainly composed of type 2 fibers, while a slow muscle is composed of type 1 fibers. The experimental method was as described by Shilveira and Fortes [29, 30].

The animals were anesthetized by the intraperitoneal injection of medetomidine (0.15 mg/kg), midazolam (2 mg/kg), and butorphanol (2.5 mg/kg). The level of anesthesia was judged by the loss of the pedal withdrawal reflex. Thereafter, their limbs were fixed on a perfusion platform and we incised the right hind limb skin and subcutaneous tissue to expose the sciatic nerve. Two platinum electrodes were attached to the hemi-lateral sciatic nerve. The insertions of the ipsilateral EDL and SOL muscle were cut and connected to a force transducer (45196A; NEC Sanei Instruments Inc., Tokyo, Japan). The resting length and stimulation voltage were adjusted to induce maximum contraction. The electrical stimulator (SEN-3301; NIHON KOHDEN, Tokyo, Japan) and isolator (SS-102 J; NIHON KOHDEN, Tokyo, Japan) were set with an impulse frequency of 1 Hz and duration every 0.5 ms. Five contractions were performed to determine the mean force (mN).

### WBT

WBT can noninvasively measure limb muscle strength. The original method evaluates the fore and hind limbs grip strength during dragging out movement [31]. Rodents are placed near an entry of a darkened tube and about to escape into it due to their nocturnal habit. Although a previous report measured the resistance of the four limbs 1 in. from the entry of the tube to its edge, in this experiment, we attached a 1-in. wire mesh on the entry of the tube to reflect the hind limb grip force, enabling the measurement of the hind limb grip force. A tail clip was connected to a force transducer and the pulling maneuver was conducted until the hind limbs were dragged out to the entry of the tube. The pulling trials were recorded 20 times to avoid the conflicting motivational influence, and the WBT10 was obtained by averaging the 10 best trials. Moreover, two investigators measured each of the 10 trials to reduce measurement bias.

### Histological analysis

After the muscle contraction measurements, the rats were deeply anesthetized with 5% sevoflurane, perfused through the ascending aorta with iced phosphate buffered saline (PBS; pH 7.2). Immediately thereafter, the EDL and SOL muscles were removed from the origin to the insertion on both sides. The EDL and SOL muscles on the right side were stained with H & E to evaluate the size of the muscle fibers in the CSA [32] and then converted into digital images (C13220–01; Hamamatsu Photonics Inc., Shizuoka, Japan). The images were analyzed by NDP view 2 (U12388–01, Hamamatsu Photonics Inc., Shizuoka, Japan) and the muscle fiber was traced to measure the CSA. The total fiber area per field at 40× magnification was collected to analyze the muscle fiber atrophy.

### Enzyme-linked immunosorbent assay (ELISA)

The EDL and SOL muscles on the residual side leg were weighed and homogenized with PBS using a multi-bead shocker (MB755U; Yasui Kikai Inc., Osaka, Japan). The homogenates were centrifuged at 5000×*g* for 5 minutes. The supernatant was stored at − 80 °C until assay. ELISA kits were used to measure MuRF-1 (OKCA01871; Aviva System Biology Inc., San Diego, CA, USA). The experiments were performed according to the manufacturer’s instructions. A microplate reader (INFINITE200; Tecan Austria Inc., Grödig, Austria) measured absorbance at 450 nm after setting the plate types. The measurements were taken three times and the average values calculated.

### Statistical methods

All results except CSA are shown as mean ± standard error of the mean (SEM) and analyzed by one-way analysis of variance followed by the Dunnett method as the post-test. Since CSA is a not a normally distributed value, it is shown as a median of the 5th – 95th percentile. CSA was analyzed by the Kruskal-Wallis test followed by the Dunn test as a post hoc analysis. Values of *p* < 0.05 were considered statistically significant. Analyses were conducted by GraphPad Prism software version 8.2.0 (El Camino Real, CA, USA).

## Data Availability

The authors confirm that the data supporting the findings of this study are available within the article.

## Abbreviations

DM: Diabetes mellitus
SHR: Spontaneously hypertensive rat
TS: Tail suspension
EDL: Extensor digitorum longus muscle
SOL: Soleus muscle
WBT: Whole body tension
CSA: Cross-sectional area
MuRF-1: Muscle RING finger-1
MAFbx: Muscle atrophy F-box
FOXO: Forkhead box O
H & E: Hematoxylin and eosin
SD: Sprague dawley
PBS: Phosphate buffered saline
ELISA: Enzyme-linked immunosorbent assay
SEM: Standard error of the mean
